# Antimicrobial activity, antibiotic susceptibility and virulence factors of Lactic Acid Bacteria of aquatic origin intended for use as probiotics in aquaculture

**DOI:** 10.1186/1471-2180-13-15

**Published:** 2013-01-24

**Authors:** Estefanía Muñoz-Atienza, Beatriz Gómez-Sala, Carlos Araújo, Cristina Campanero, Rosa del Campo, Pablo E Hernández, Carmen Herranz, Luis M Cintas

**Affiliations:** 1 Grupo de Seguridad y Calidad de los Alimentos por Bacterias Lácticas, Bacteriocinas y Probióticos (Grupo SEGABALBP) Departamento de Nutrición, Bromatología y Tecnología de los Alimentos, Facultad de Veterinaria, Universidad Complutense de Madrid, Madrid, 28040, Spain; 2 Centro de Genética e Biotecnologia, Universidade de Trás-os-Montes e Alto Douro, Vila Real, Portugal; 3 Servicio de Microbiología, Hospital Universitario Ramón y Cajal, Madrid, 28034, Spain

**Keywords:** Lactic Acid Bacteria, Aquatic animals, Aquaculture probiotics, Anti-fish pathogens activity, Antibiotic resistance and virulence factors, Qualified Presumption of Safety

## Abstract

**Background:**

The microorganisms intended for use as probiotics in aquaculture should exert antimicrobial activity and be regarded as safe not only for the aquatic hosts but also for their surrounding environments and humans. The objective of this work was to investigate the antimicrobial/bacteriocin activity against fish pathogens, the antibiotic susceptibility, and the prevalence of virulence factors and detrimental enzymatic activities in 99 Lactic Acid Bacteria (LAB) (59 enterococci and 40 non-enterococci) isolated from aquatic animals regarded as human food.

**Results:**

These LAB displayed a broad antimicrobial/bacteriocin activity against the main Gram-positive and Gram-negative fish pathogens. However, particular safety concerns based on antibiotic resistance and virulence factors were identified in the genus *Enterococcus* (86%) (*Enterococcus faecalis*, 100%; *E. faecium,* 79%). Antibiotic resistance was also found in the genera *Weissella* (60%), *Pediococcus* (44%), *Lactobacillus* (33%), but not in leuconostocs and lactococci. Antibiotic resistance genes were found in 7.5% of the non-enterococci, including the genera *Pediococcus* (12.5%) and *Weissella* (6.7%). One strain of both *Pediococcus pentosaceus* and *Weissella cibaria* carried the erythromycin resistance gene *mef*(A/E), and another two *P. pentosaceus* strains harboured *lnu*(A) conferring resistance to lincosamides. Gelatinase activity was found in *E. faecalis* and *E. faecium* (71 and 11%, respectively), while a low number of *E. faecalis* (5%) and none *E. faecium* exerted hemolytic activity. None enterococci and non-enterococci showed bile deconjugation and mucin degradation abilities, or other detrimental enzymatic activities.

**Conclusions:**

To our knowledge, this is the first description of *mef*(A/E) in the genera *Pediococcus* and *Weissella*, and *lnu*(A) in the genus *Pediococcus*. The *in vitro* subtractive screening presented in this work constitutes a valuable strategy for the large-scale preliminary selection of putatively safe LAB intended for use as probiotics in aquaculture.

## Background

Aquaculture has the potential to make a significant contribution to the increasing demand for aquatic food in most world regions; however, in order to achieve this goal, the sector will have to face significant challenges, including the production intensification, the disease control and the prevention of the environmental deterioration [1]. In fish farming, the widespread use of antibiotics as prophylactic and therapeutic agents to control bacterial diseases has been associated with the emergence of antibiotic resistance in bacterial pathogens and with the alteration of the microbiota of the aquaculture environment [2,3]. This resulted in the ban of antibiotic usage as animal growth promoters in Europe and stringent worldwide regulations on therapeutical antibiotic applications. This scenario has led to an evergrowing interest in the search and development of alternative strategies for disease control, within the frame of good husbandry practices, including adequate hygiene conditions, vaccination programmes and the use of probiotics, prebiotics and immunostimulants [4-6]. Recently, novel strategies to control bacterial infections in aquaculture have emerged, such as specific killing of pathogenic bacteria by bacteriophages, growth inhibition of pathogen by short-chain fatty acids and polyhydroxyalkanoates, and interference with the regulation of virulence genes (quorum sensing disruption), which have been reviewed by Defoirdt *et al.*[7]. With regard to probiotics, they are defined as live microbial adjuncts which have a beneficial effect on the host by: (i) modifying the host-associated or ambient microbial community; (ii) improving feed use or enhancing its nutritional value; (iii) enhancing the host response towards disease; and/or (iv) improving its environment [8]. To date, most probiotics proposed as biocontrollers and bioremediation agents for aquaculture belong to the LAB group (mainly to the genera *Lactobacillus*, *Lactococcus*, *Leuconostoc*, *Enterococcus* and *Carnobacterium*), to the genera *Vibrio*, *Bacillus*, and *Pseudomonas* or to the species *Saccharomyces cerevisiae*[8,9]. Recently, a probiotic culture (Bactocell®, *Pediococcus acidilactici* CNCM MA18/5 M) has been authorized for the first time for use in aquaculture in the European Union.

According to the FAO/WHO [10], the development of commercial probiotics requires their unequivocal taxonomic identification, as well as their *in vitro* and *in vivo* functional characterization and safety assessment. In Europe, the European Food Safety Agency (EFSA) proposed a system for a pre-market safety assessment of selected groups of microorganisms used in food/feed and the production of food/feed additives leading to a Qualified Presumption of Safety (QPS) status [11-13]. The QPS approach propose that the safety assessment of a defined taxonomic group could be made based on establishing taxonomic identity, body of knowledge, possible pathogenicity and commercial end use. According to the EFSA approach [13], most LAB species are included in the QPS list and, therefore, demonstration of their safety only requires confirmation of the absence of determinants of resistance to antibiotics of human and veterinary clinical significance. However, in the case of enterococci, a more thorough, strain-specific evaluation is required to assess the risk associated to their intentional use in the food chain. In this work, we present the antimicrobial activity against fish pathogens and the *in vitro* safety assessment beyond the QPS approach of a collection of 99 LAB belonging to the genera *Enterococcus*, *Lactobacillus*, *Lactococcus*, *Leuconostoc*, *Pediococcus* and *Weissella*, previously isolated from aquatic animals regarded as human food [14] and intended for use as probiotics in aquaculture.

## Results

### Direct antimicrobial activity of the 99 LAB of aquatic origin

The 99 LAB strains isolated from fish, seafood and fish products displayed direct antimicrobial activity against, at least, four of the eight tested indicator microorganisms (Table 1). The most sensitive indicators were *Listonella anguillarum* CECT4344, *Ls. anguillarum* CECT7199 and *Aeromonas hydrophila* CECT5734, followed by *Lactococcus garvieae* JIP29-99, *Streptococcus iniae* LMG14521 and *Streptococcus agalactiae* CF01173. On the contrary, *Photobacterium damselae* CECT626 and *Vibrio alginolyticus* CECT521 were the less sensitive indicator microorganisms.

**Table 1:** Origin and direct antimicrobial activity against fish pathogens of LAB isolated from aquatic animals

**Origin**	**Strain**		**Indicator microorganisms**^**a**^							
			***Lactococcus garvieae *JIP29-99**	***Streptococcus agalactiae *CF01173**	***Streptococcus iniae *LMG14521**	***Aeromonas hydrophila *CECT5734**	***Listonella anguillarum *CECT4344**	***Ls. anguillarum *CECT7199**	***Photobacterium damselae *CECT626**	***Vibrio alginolyticus *CECT521**
Albacore (*Thunnus alalunga*)	*Enterococcus faecium*	BNM58	+	+	+	++	++	+++	+	-
	*Weissella cibaria*	BNM69	+	+	+	+++	+++	+++	-	-
Atlantic salmon (*Salmo salar*)	*Enterococcus faecalis*	SMF10	+	+	+	++	+++	++	-	+
		SMF28	+	+	++	++	+++	+	-	+
		SMF37	+	+	+	+	++	+++	-	+
		SMF69	+	+	++	++	+++	+++	+	+
		SMM67	+	+	++	++	+++	+++	-	-
		SMM70	+	+	+	+	+++	+++	-	-
	*E. faecium*	SMA1	+	+	+	++	++	+++	+	-
		SMA7	+	+	+	+	++	+++	+	+
		SMA8	+	+	+	++	++	+++	+	+
		SMA101	+	+	+	++	+++	++	+	+
		SMA102	+	+	+	++	+++	+	+	+
		SMA310	++	+	+	++	+++	++	+	+
		SMA320	++	+	+	++	++	+++	+	+
		SMA361	+	+	+	++	++	+++	+	+
		SMA362	+	+	+	++	++	+++	+	-
		SMA384	+	+	+	++	++	+++	+	-
		SMA389	+	+	+	++	++	+++	-	+
		SMF8	+	+	++	++	++	++	+	-
		SMF39	+	+	++	++	++	+++	+	+
	*Lactobacillus sakei* subsp. c*arnosus* (*Lb. carnosus*)	SMA17	+	-	+	++	+++	+++	-	-
	*Lactococcus lactis* subsp. *cremoris* (*L. cremoris*)	SMF110	+	+	+	+	+++	+++	+	+
		SMF161	+	+	+	++	+++	+++	+	++
		SMF166	+	+	+	++	++	+++	+	++
	*Leuconostoc mesenteroides* subsp. *cremoris* (*Lc. cremoris*)	SMM69	+	+	+	++	+++	+++	-	-
	*Pediococcus pentosaceus*	SMF120	++	++	++	++	+++	+++	-	+
		SMF130	++	+	++	++	+++	+++	-	+
		SMM73	++	+	+	+++	+++	+++	+	++
	*W. cibaria*	SMA14	++	+	+	++	+++	+++	+	++
		SMA25	+	+	+	+++	+++	+++	-	-
Cod (*Gadus morhua*)	*E. faecalis*	BCS27	++	++	++	++	+++	+++	-	-
		BCS32	+	+	+	+	++	+++	-	+
		BCS53	+	++	+	+	+++	+++	+	-
		BCS67	+	+	-	++	+++	++	-	+
		BCS72	+	+	+	++	+++	+++	+	-
		BCS92	+	+	+	++	+++	++	+	+
	*E. faecium*	BCS59	++	+	++	++	+++	+++	-	+
		BCS971	+	+	+	+	+++	+++	-	+
		BCS972	+	+	+	+	+++	+++	-	+
	*Lactobacillus curvatus* subsp. *curvatus* (*Lb. curvatus*)	BCS35	-	-	+	++	+++	+++	-	-
	*Lc. cremoris*	BCS251	+	+	++	+	+++	+++	-	+
		BCS252	+	+	++	+	+++	+++	-	+
	*P. pentosaceus*	BCS46	++	+	++	+++	+++	+++	-	+
	*W. cibaria*	BCS50	++	+	++	++	+++	+++	-	+
Common cockle (*Cerastoderma edule*)	*E. faecium*	B13	+	+	++	++	+++	+++	-	-
		B27	+	+	+	++	+++	++	+	+
	*Lb. carnosus*	B43	+	+	+	++	+++	+++	-	-
	*P. pentosaceus*	B5	++	+	++	++	+++	+++	-	-
		B11	++	+	++	+++	+++	+++	+	-
		B41	++	++	++	+++	+++	+++	+	++
		B260	++	+	++	++	+++	+++	-	++
	*W. cibaria*	B4620	++	+	++	++	+++	+++	-	++
Common ling (*Molva molva*)	*E. faecium*	MV5	+	+	+	++	++	+++	+	+
Common octopus (*Octopus vulgaris*)	*E. faecalis*	P77	++	+	++	++	+++	+++	-	+
	*E. faecium*	P68	++	+	+++	++	+++	+++	-	+
		P623	+	+	+	+	+++	++	-	+
	*P. pentosaceus*	P63	++	+	++	+++	+++	+++	-	+
		P621	++	+	++	+	+++	+++	-	+
	*W. cibaria*	P38	++	++	++	++	+++	+++	-	+
		P50	++	+	+	++	+++	+++	-	+
		P61	++	+	+	++	+++	+++	-	-
		P64	++	+	+	+++	+++	+++	+	++
		P69	++	+	+	++	+++	+++	+	++
		P71	+	+	++	++	+++	+++	+	+
		P73	++	++	++	++	+++	+++	-	+
		P622	++	++	++	+	+++	+++	+	+
European seabass (*Dicentrarchus labrax*)	*E. faecium*	LPP29	+	+	+	+	++	+++	+	-
	*P. pentosaceus*	LPM78	++	+	++	++	+++	+++	-	-
		LPM83	++	+	++	++	+++	+++	-	-
		LPP32	++	++	++	++	+++	+++	-	+
		LPV46	++	+	++	++	+++	+++	-	+
		LPV57	++	+	++	+++	+++	+++	-	-
European squid (*Loligo vulgaris*)	*E. faecium*	CV1	+	+	+	+	+++	+++	-	+
		CV2	++	+	+	+	+++	++	+	+
Megrim (*Lepidorhombus boscii*)	*E. faecalis*	GM22	-	-	+	++	++	+++	+	++
		GM26	-	-	+	+	++	++	+	-
		GM33	-	-	++	+	++	+++	+	-
	*E. faecium*	GM23	+	+	+	++	++	+++	+	+
		GM29	++	++	+	++	++	+++	+	+
		GM351	-	-	+	+	++	++	+	-
		GM352	++	+	+	++	++	+++	+	+
Norway lobster (*Nephrops norvegicus*)	*E. faecalis*	CGM16	++	+	++	++	+++	+++	-	+
		CGM156	+	+	++	++	+++	+++	-	-
		CGM1514	+	+	+	++	+++	++	+	+
		CGV67	++	+	+	+	+++	+++	+	+
	*E. faecium*	CGM171	+	+	+	+	+++	+++	+	+
		CGM172	+	+	+	+	+++	+++	+	+
Rainbow trout (*Oncorhynchus mykiss*)	*E. faecium*	TPM76	+	+	+	+	++	+++	+	+
		TPP2	+	+	+	+	++	+++	+	+
	*P. pentosaceus*	TPP3	++	+	+	++	+++	+++	-	++
Sardine (*Sardina pilchardus*)	*E. faecalis*	SDP10	+	+	+	+	+++	+++	-	+
	*W. cibaria*	SDM381	++	+	++	++	+++	+++	-	-
		SDM389	+	+	++	++	+++	+++	-	-
Swimcrab (*Necora puber*)	*E. faecium*	NV50	+	+	+	++	++	++	+	-
		NV51	++	+	+	+	++	++	+	++
		NV52	++	+	+	+	++	+++	+	+
		NV54	++	+	+	+	++	+++	+	+
		NV56	++	+	+	++	++	++	+	-

^a^Direct antimicrobial activity was determined by a SOAT and the scores reflect different degrees of growth inhibition (diameter in mm); -, no inhibition; +, 3–5 mm inhibition zone; ++, 6–9 mm inhibition zone; +++, ≥10 mm inhibition zone.

### Preliminary safety evaluation of enterococci: presence of virulence factors, production of gelatinase and hemolysin and antibiotic susceptibility

Concerning *E. faecalis*, most of the strains (20 strains, 95%) harboured, at least, one relevant virulence factor: *efaAfs* (95%), *gelE* (71%), or *agg* (67%) genes (Table 2). A positive gelatinase reaction was found in 15 *E. faecalis* strains (71%) which harboured *gelE*, from which 12 also harboured *agg* gene. Only one *E. faecalis* strain (*E. faecalis* SDP10) (5%), harbouring *cylL*_*L*_*-cylL*_*S*_*-cylM*, exerted hemolytic activity, while none of the strains amplified *hyl* or *esp* genes. With regard to *E. faecium*, 20 strains (53%) harboured, at least, one relevant virulence factor: *efaAfs* (45%), *gelE* (24%) or *agg* (8%), but only 4 strains (11%) exerted gelatinase activity. None of the *E. faecium* strains exerted hemolytic activity nor amplified *hyl* or *esp* genes. The results of the antibiotic susceptibility tests revealed that 39 enterococccal strains (66%) displayed acquired antibiotic resistance to antibiotics other than penicillin G, chloramphenicol and high-level gentamicin. In this respect, 13 *E. faecalis* strains (62%) showed acquired resistance to (i) second generation quinolones (ciprofloxacin and/or norfloxacin) (12 strains, 57%), (ii) rifampicin (5 strains, 24%), (iii) nitrofurantoin (5 strains, 24%), (iv) glycopeptides (vancomycin and teicoplanin) (4 strains, 19%), and/or (v) erythromycin (1 strain, 5%). However, 26 *E. faecium* strains (68%), including 17 strains that encode virulence factors and nine strains without these traits, displayed acquired resistance to (i) erythromycin (14 strains, 37%), (ii) nitrofurantoin (11 strains, 29%), (iii) second generation quinolones (ciprofloxacin and/or norfloxacin) (10 strains, 26%), (iv) rifampicin (4 strains, 11%), (v) tetracycline (2 strains, 5%), and/or (vi) glycopeptides (vancomycin and teicoplanin) (1 strain, 3%). Moreover, multiple antibiotic resistance (two to six antibiotics) was found in *E. faecalis* (10 strains, 48%) and, to a lesser extent, in *E. faecium* (12 strains, 32%) (Table 2). According to the results above, 21 *E. faecalis* strains were discarded for further studies based on the presence of virulence factors (8 strains, 38%), acquired antibiotic resistance (1 strain, 5%) or both (12 strains, 57%). Regarding *E. faecium* strains, 29 (76%) were dropped from further screening based on acquired antibiotic resistance (9 strains, 24%), the presence of virulence factors (3 strains, 8%) or both (17 strains, 45%).

**Table 2:** Preliminary safety evaluation of enterococci

***Enterococcus *spp.**	**Strain**	**Virulence Factors**		**Antibiotic resistance phenotype**^**c**^
		**Genotype**^**a**^	**Phenotype**^**b**^	
*E. faecalis*	SMF10	*efaAfs*^+^, *gelE*^+^, *agg*^+^	GelE^+^, Hly^-^	CIP, NOR
	SMF28	*efaAfs*^+^, *gelE*^+^	GelE^+^, Hly^-^	CIP, NOR
	SMF37	*efaAfs*^+^, *gelE*^+^, *agg*^+^	GelE^+^, Hly^-^	-
	SMF69	*efaAfs*^+^, *gelE*^+^, *agg*^+^	GelE^+^, Hly^-^	CIP, RIF
	SMM67	n.d.	GelE^-^, Hly^-^	CIP, NIT, NOR, TEC, VAN
	SMM70	*efaAfs*^+^, *gelE*^+^	GelE^+^, Hly^-^	ERY, NIT
	BCS27	*efaAfs*^+^, *gelE*^+^, *agg*^+^	GelE^+^, Hly^-^	CIP, NIT, NOR, RIF, TEC, VAN
	BCS32	*efaAfs*^+^, *gelE*^+^, *agg*^+^	GelE^+^, Hly^-^	NOR
	BCS53	*efaAfs*^+^, *gelE*^+^, *agg*^+^	GelE^+^, Hly^-^	-
	BCS67	*efaAfs*^+^	GelE^-^, Hly^-^	CIP
	BCS72	*efaAfs*^+^, *agg*^+^	GelE^-^, Hly^-^	-
	BCS92	*efaAfs*^+^	GelE^-^, Hly^-^	-
	P77	*efaAfs*^+^, *gelE*^+^	GelE^+^, Hly^-^	NIT, NOR, RIF, TEC, VAN
	GM22	*efaAfs*^+^, *gelE*^+^, *agg*^+^	GelE^+^, Hly^-^	CIP, NOR
	GM26	*efaAfs*^+^, *gelE*^+^, *agg*^+^	GelE^+^, Hly^-^	-
	GM33	*efaAfs*^+^, *gelE*^+^, *agg*^+^	GelE^+^, Hly^-^	-
	CGM156	*efaAfs*^+^	GelE^-^, Hly^-^	CIP, NIT, NOR, RIF, TEC, VAN
	CGM1514	*efaAfs*^+^, *agg*^+^	GelE^-^, Hly^-^	-
	CGM16	*efaAfs*^+^, *gelE*^+^, *agg*^+^	GelE^+^, Hly^-^	CIP, NOR, RIF
	CGV16	*efaAfs*^+^, *gelE*^+^, *agg*^+^	GelE^+^, Hly^-^	NOR
	SDP10	*efaAfs*^+^, *gelE*^+^, *agg*^*+*^, *cylL*_*L*_*L*_*S*_^*+*^*, cylL*_*L*_*L*_*S*_*M*^*+*^	GelE^+^, Hly^+^	-
*E. faecium*	BNM58	n.d.	GelE^-^, Hly^-^	-
	SMA1	n.d.	GelE^-^, Hly^-^	CIP
	SMA7	n.d.	GelE^-^, Hly^-^	-
	SMA8	n.d.	GelE^-^, Hly^-^	-
	SMA101	n.d.	GelE^-^, Hly^-^	ERY, NIT
	SMA102	*efaAfs*^+^	GelE^-^, Hly^-^	ERY, NIT
	SMA310	n.d.	GelE^-^, Hly^-^	ERY, NIT
	SMA320	*efaAfs*^+^	GelE^-^, Hly^-^	ERY, NIT
	SMA361	*efaAfs*^+^	GelE^-^, Hly^-^	ERY
	SMA362	n.d.	GelE^-^, Hly^-^	ERY, NIT
	SMA384	*gelE*^+^	GelE^-^, Hly^-^	NIT
	SMA389	*gelE*^+^	GelE^-^, Hly^-^	CIP, NIT, NOR
	SMF8	n.d.	GelE^-^, Hly^-^	-
	SMF39	*efaAfs*^+^, *gelE*^+^	GelE^-^, Hly^-^	-
	BCS59	n.d.	GelE^-^, Hly^-^	NIT
	BCS971	n.d.	GelE^-^, Hly^-^	ERY
	BCS972	n.d.	GelE^-^, Hly^-^	ERY
	B13	*gelE*^+^	GelE^+^, Hly^-^	CIP
	B27	*efaAfs*^+^, *gelE*^+^	GelE^+^, Hly^-^	CIP
	MV5	*efaAfs*^+^, *gelE*^+^, *agg*^+^	GelE^-^, Hly^-^	CIP, NIT
	P68	*efaAfs*^+^, *gelE*^+^, *cylL*_*L*_*L*_*S*_^*+*^	GelE^+^, Hly^-^	CIP, NIT, NOR, RIF, TEC, VAN
	P623	*efaAfs*^+^	GelE^-^, Hly^-^	ERY
	LPP29	n.d.	GelE^-^, Hly^-^	-
	CV1	n.d.	GelE^-^, Hly^-^	-
	CV2	n.d.	GelE^-^, Hly^-^	-
	GM23	*efaAfs*^+^	GelE^-^, Hly^-^	CIP, NOR, RIF, TET
	GM29	*efaAfs*^+^, *gelE*^+^, *cylL*_*L*_*L*_*S*_^*+*^	GelE^-^, Hly^-^	CIP, NOR, RIF
	GM351	*efaAfs*^+^, *gelE*^+^, *agg*^+^	GelE^+^, Hly^-^	CIP, NOR
	GM352	*efaAfs*^+^	GelE^-^, Hly^-^	CIP, NIT, NOR, RIF, TET
	CGM171	n.d.	GelE^-^, Hly^-^	ERY
	CGM172	*efaAfs*^+^	GelE^-^, Hly^-^	ERY
	TPM76	n.d.	GelE^-^, Hly^-^	-
	TPP2	n.d.	GelE^-^, Hly^-^	-
	NV50	*efaAfs*^+^, *agg*^+^	GelE^-^, Hly^-^	-
	NV51	*efaAfs*^+^	GelE^-^, Hly^-^	ERY
	NV52	n.d.	GelE^-^, Hly^-^	ERY
	NV54	*efaAfs*^+^	GelE^-^, Hly^-^	ERY
	NV56	*efaAfs*^+^	GelE^-^, Hly^-^	-

^a^n.d., not detected.

^b^GelE and Hly refer to gelatinase and cytolysin/hemolysin activity, respectively.

^c^Abbreviation of antibiotics: CIP, ciprofloxacin; ERY, erythromycin; NIT, nitrofurantoin; NOR, norfloxacin; RIF, rifampicin; TEC, teicoplanin; TET, tetracycline; VAN, vancomycin.

### Extracellular antimicrobial activity of the 49 pre-selected LAB

The antimicrobial activity of supernatants from the 49 pre-selected LAB (9 *E. faecium* selected based on their preliminary safety assessment and 40 non-enterococcal strains) with direct antimicrobial activity against fish pathogens was assayed against three indicator microorganisms by an ADT (Table 3). In this regard, 24 (49%) and 10 (20%) strains displayed extracellular antimicrobial activity in their supernatants and/or 20-fold concentrated supernatants against *Pediococcus damnosus* CECT4797 and *L. garvieae* JIP 29–99, respectively, but none of the strains inhibited the Gram-negative strain *A. hydrophila* CECT5734. Interestingly, the antimicrobial activity of the respective supernatants was sensitive to proteinase K treatment, but was not affected by the heat treatment, revealing the proteinaceous nature and heat stability of the secreted antimicrobial compounds (i.e., heat-stable bacteriocins). The 24 LAB strains secreting bacteriocins into the liquid growth medium belong to the species *P*. *pentosaceus* (15 strains), *E. faecium* (8 strains)*,* and *Lb*. *curvatus* (1 strain).

**Table 3:** **Extracellular antimicrobial activity of the 49 pre-selected LAB**^**a**^

**LAB species**^**b**^	**Strain**	**Indicator microorganisms**					
		***P. damnosus *CECT4797**		***L. garvieae *JIP29-99**		***A. hydrophila *CECT5734**	
		**S**	**CS**	**S**	**CS**	**S**	**CS**
**Enterococci**							
*E. faecium*	BNM58	22.4	26.8	14.0	15.0	-	-
	SMA7	-	-	-	-	-	-
	SMA8	19.0	19.6	9.4	10.2	-	-
	SMF8	19.0	21.8	10.3	10.8	-	-
	LPP29	20.5	24.4	12.6	13.1	-	-
	CV1	15.0	19.2	-	-	-	-
	CV2	19.8	23.7	12.7	11.4	-	-
	TPM76	17.0	21.2	-	8.7	-	-
	TPP2	19.7	23.5	12.8	12.4	-	-
**Non-enterococci**							
*Lb. curvatus*	BCS35	18.2	24.7	-	-	-	-
*P. pentosaceus*	SMF120	-	-	-	-	-	-
	SMF130	7.4	9.7	-	-	-	-
	SMM73	-	9.5	-	-	-	-
	BCS46	-	9.4	-	-	-	-
	B5	8.1	9.0	-	-	-	-
	B11	-	9.0	-	-	-	-
	B41	7.3	11.7	-	-	-	-
	B260	7.3	10.6	-	-	-	-
	P63	-	9.8	-	-	-	-
	P621	-	10.5	-	-	-	-
	LPM78	-	8.3	-	-	-	-
	LPM83	7.9	11.0	-	-	-	-
	LPP32	8.5	11.3	-	8.9	-	-
	LPV46	8.2	11.3	-	8.2	-	-
	LPV57	7.6	10.5	-	-	-	-
	TPP3	9.0	11.7	7.5	9.2	-	-

^a^Antimicrobial activity (mm) of supernatants (S) and 20-fold concentrated supernatants (CS) as determined by an ADT.

^*b*^*Lb. carnosus*, *L. cremoris*, *Lc. cremoris* and *W. cibaria* strains did not show extracellular antimicrobial activity against any of the tested indicator microorganisms.

### *In vitro* safety assessment of the 49 pre-selected LAB

The 49 pre-selected LAB were further submitted to a comprehensive safety assessment by different *in vitro* tests.

### Hemolysin production, bile salts deconjugation and mucin degradation

None of the non-enterococcal strains showed hemolytic activity, similarly as found for the 9 enterococci. Moreover, bile salts deconjugation and mucin degradation abilities were not found in any of the tested strains.

### Enzymatic activities

The results of the analysis of enzymatic activity profiles of the tested LAB are shown in Table 4. None of the strains showed lipolytic activity, except *E. faecium* LPP29, TPM76, SMA7, and SMF8 which produced esterase (C4) and esterase lipase (C8). Moreover, none of the LAB strains showed protease activity (trypsin and α-chymotrypsin). Nevertheless, peptidase activity (leucine, valine or cystine arylamidase) was found in all the species. All strains showed acid phosphatase (except *E. faecium* TPM76 and *Lc. cremoris*) and naphthol-AS-BI-phosphohydrolase activities, but none displayed alkaline phosphatase activity. β-Galactosidase was found in most species (but not in all strains) except *Lb. curvatus* and *L*. *cremoris*. However, α-glucosidase was only found in the three *Lc. cremoris* strains. β-Glucosidase and N-acetyl-β-glucosaminidase activities were observed in most *E. faecium*, *Lactobacillus* spp., *L. cremoris*, and *P. pentosaceus* strains, but only in two *W. cibaria* strains, while the three *Lc. cremoris* strains showed β-glucosidase but lacked N-acetyl-β-glucosaminidase activity. On the other hand, α-galactosidase, β-glucuronidase, α-mannosidase, and α-fucosidase activities were not detected in any of the tested LAB strains.

**Table 4:** **Enzymatic activity profiles of the 49 pre-selected LAB**^**a**^

**Species**	**Strain**	**Esterase (C4)**	**Esterase lipase (C8)**	**Leucine arylamidase**	**Valine arylamidase**	**Cystine arylamidase**	**Acid phosphatase**	**Naphthol-AS-BI- phosphohydrolase**	**β-Galactosidase**	**α-Glucosidase**	**β-Glucosidase**	**N-acetyl-β-glucosaminidase**
**Enterococci**												
*E. faecium*	BNM58	0	0	≥40	10	10	20	10	0	0	0	0
	SMA7	20	20	≥40	30	20	30	10	0	0	0	0
	SMA8	0	0	≥40	≥40	5	5	5	5	0	20	≥40
	SMF8	5	5	10	5	5	20	10	0	0	30	0
	LPP29	10	10	30	5	20	10	10	0	0	0	0
	CV1	0	0	≥40	≥40	5	10	20	20	0	30	≥40
	CV2	0	0	≥40	≥40	10	10	20	0	0	10	≥40
	TPM76	30	10	20	0	0	0	10	10	0	0	0
	TPP2	0	0	≥40	20	10	10	10	5	0	30	0
**Non-enterococci**												
*Lb. carnosus*	SMA17	0	0	≥40	≥40	0	30	20	30	0	30	30
	B43	0	0	≥40	≥40	0	5	5	10	0	0	0
*Lb. curvatus*	BCS35	0	0	≥40	10	5	10	20	0	0	5	10
*L. cremoris*	SMF110	0	0	≥40	≥40	0	20	20	0	0	30	30
	SMF161	0	0	20	0	5	≥40	20	0	0	0	0
	SMF166	0	0	≥40	≥40	0	20	20	0	0	10	10
*Lc. cremoris*	SMM69	0	0	10	0	0	0	10	≥40	30	≥40	0
	BCS251	0	0	5	0	0	0	5	20	20	10	0
	BCS252	0	0	10	0	0	0	10	30	20	10	0
*P. pentosaceus*	SMF120	0	0	≥40	≥40	20	≥40	≥40	0	0	20	20
	SMF130	0	0	≥40	≥40	20	30	≥40	20	0	≥40	≥40
	SMM73	0	0	≥40	30	10	20	30	20	0	30	≥40
	BCS46	0	0	≥40	≥40	5	20	30	30	0	≥40	≥40
	B5	0	0	30	≥40	10	10	20	10	0	30	≥40
	B11	0	0	≥40	30	0	5	20	0	0	30	≥40
	B41	0	0	30	≥40	0	5	20	5	0	20	≥40
	B260	0	0	≥40	≥40	10	20	30	0	0	20	30
	P63	0	0	≥40	≥40	5	20	20	30	0	30	≥40
	P621	0	0	≥40	≥40	0	5	30	0	0	30	≥40
	LPM78	0	0	30	30	5	10	20	20	0	30	≥40
	LPM83	0	0	30	30	5	10	20	30	0	10	≥40
	LPP32	0	0	≥40	≥40	5	5	20	0	0	30	≥40
	LPV46	0	0	≥40	≥40	5	20	30	5	0	30	30
	LPV57	0	0	≥40	≥40	5	20	30	30	0	≥40	≥40
	TPP3	0	0	≥40	≥40	5	5	5	10	0	0	0
*W. cibaria*	BNM69	0	0	0	0	0	30	10	30	0	0	0
	SMA14	0	0	0	0	0	20	5	10	0	0	0
	SMA25	0	0	≥40	≥40	0	30	20	≥40	0	30	30
	BCS50	0	0	0	0	0	30	20	30	0	0	0
	B4620	0	0	20	20	0	30	20	30	0	5	5
	P38	0	0	0	0	0	≥40	20	≥40	0	0	0
	P50	0	0	0	0	0	≥40	20	0	0	0	0
	P61	0	0	0	0	0	20	10	0	0	0	0
	P64	0	0	0	0	0	30	10	0	0	0	0
	P69	0	0	0	0	0	≥40	20	≥40	0	0	0
	P71	0	0	0	0	0	≥40	10	0	0	0	0
	P73	0	0	0	0	0	30	20	30	0	0	0
	P622	0	0	0	0	0	≥40	10	0	0	0	0
	SDM381	0	0	10	5	0	20	10	30	0	0	0
	SDM389	0	0	0	0	0	≥40	20	≥40	0	0	0

^a^Enzymatic activities determined by an APIZYM test. Relative activity between 0 and ≥ 40 nmol.

### Antibiotic susceptibility determined by the broth microdilution test

The distribution of MICs of the tested antibiotics is summarized in Tables 5 and 6. Microbiological breakpoints for ampicillin, vancomycin, gentamicin, kanamycin, streptomycin, erythromycin, clindamycin, tetracycline, and chloramphenicol reported by the FEEDAP document on the assessment of bacterial products used as feed additives in relation to antibiotic resistance [15] were used to categorise the 49 LAB as susceptible or resistant strains. In this document, the genus *Weissella*, which is considered a group of heterofermentative *Leuconostoc*-like LAB [16], is not included. For this reason, the respective MICs were interpreted by using the breakpoints given for the genus *Leuconostoc*. Besides, due to the lack of microbiological breakpoints for penicillin and linezolid on the FEEDAP document, we interpreted our results on these antibiotics according to the cut-off levels proposed by Klare *et al.*[17] for pediococci, namely 1 and 2 mg/L for penicillin and linezolid, respectively. According to our results, the percentages of strains showing antibiotic resistance in the genera *Weissella*, *Pediococcus*, *Lactobacillus* and *Enterococcus* were 60, 44, 33 and 11%, respectively, while none of the leuconostocs and lactococci showed this phenotype. In summary, 97.5% of the 40 non-enterococal strains resulted susceptible to ampicillin, 100% to gentamicin, 72.5% to kanamycin, 100% to streptomycin, 95% to erythromycin, 87.5% to clindamycin, 95% to tetracycline, and 100% to chloramphenicol. For vancomycin, it is known that facultative and obligate heterofermentative *Lactobacillus, Pediococcus* spp. and *Leuconostoc* spp. are intrinsically resistant. In contrast, the three lactococci were clearly susceptible to these antibiotics, showing a MIC of 0.5 mg/L. On the other hand, according to the cut-off values proposed by Klare *et al.*[17], 93% of *P. pentosaceus* strains were susceptible to penicillin and linezolid. With regard to *E. faecium*, all the tested strains were susceptible to ampicillin, vancomycin, gentamicin, kanamycin, streptomycin, tetracycline, chloramphenicol, and erythromycin except *E. faecium* BNM58 against the latter antibiotic (MIC = 8 mg/L). Moreover, multiple antibiotic resistance (three antibiotics) was only detected in *P. pentosaceus* LPM78 (6.2%) and *W. cibaria* SMA25 (6.7%).

**Table 5:** MICs distribution of 10 antibiotics for the 9 enterococcal strains

**Antibiotics**	**Number of strains with the indicated MIC (mg/L)**^**a**^																**EFSA breakpoints (mg/L)**^**b**^
	**0.06**	**0.12**	**0.25**	**0.5**	**1**	**2**	**4**	**8**	**16**	**32**	**64**	**128**	**256**	**512**	**1024**	**2048**	
Ampicillin			5	3	1												**2**
Vancomycin				9													**4**
Gentamicin					4		5										**32**
Kanamycin								1		2	4	2					**1024**
Streptomycin							1		3	5							**128**
Erythromycin			5				3	**1**									**4**
Tetracycline					9												**4**
Chloramphenicol							8	1									**16**
Linezolid						9											**n.a.**
Narasin			1	8													**n.a.**

^a^MICs determined by a VetMIC test. The antibiotic dilution ranges were: 0.25-32 mg/L (ampicillin), 1-128 mg/L (vancomycin), 2-256 mg/L (gentamicin), 16-2048 mg/L (kanamycin), 8-1024 mg/L (streptomycin), 0.5-64 mg/L (erythromycin, tetracycline and chloramphenicol), 0.25-16 mg/L (linezolid) and 0.12-16 (narasin). MICs which exceeded the upper or lower limit of the tested range are listed in the next dilution series. MICs higher than the EFSA breakpoints are indicated in bold.

^b^LAB with MICs higher than the EFSA breakpoints are considered as resistant strains [15]. n.a., not available.

**Table 6:** MICs distribution of 15 antibiotics for the 40 non-enterococcal strains

**Antibiotics**	**Species (no. of tested isolates)**	**Number of strains with the indicated MIC (mg/L)**^**a**^																		**EFSA breakpoints (mg/L)**^**b**^
		**0.016**	**0.03**	**0.06**	**0.12**	**0.25**	**0.5**	**1**	**2**	**4**	**8**	**16**	**32**	**64**	**128**	**256**	**512**	**1024**	**2048**	
Ampicillin	*Lb. carnosus* (2)									1	**1**									**4**
	*Lb. curvatus* (1)						1													**4**
	*L. cremoris* (3)				1	2														**2**
	*Lc. cremoris* (3)				1	2														**2**
	*P. pentosaceus* (16)								15	1										**4**
	*W. cibaria* (15)						15													**n.a.**
Vancomycin	*Lb. carnosus* (2)										2									**n.r.**
	*Lb. curvatus* (1)											1								**n.r.**
	*L. cremoris* (3)						3													**4**
	*Lc. cremoris* (3)															3				**n.r.**
	*P. pentosaceus* (16)															16				**n.r.**
	*W. cibaria* (15)															15				**n.a.**
Gentamicin	*Lb. carnosus* (2)						1		1											**16**
	*Lb. curvatus* (1)									1										**16**
	*L. cremoris* (3)					3														**32**
	*Lc. cremoris* (3)					3														**16**
	*P. pentosaceus* (16)					1		1	9	3	2									**16**
	*W. cibaria* (15)					6		7	1		1									**n.a.**
Kanamycin	*Lb. carnosus* (2)								1		1									**64**
	*Lb. curvatus* (1)											1								**64**
	*L. cremoris* (3)								2	1										**64**
	*Lc. cremoris* (3)										1	2								**16**
	*P. pentosaceus* (16)										1			13	**2**					**64**
	*W. cibaria* (15)									1	1	4	4	4	1					**n.a.**
Streptomycin	*Lb. carnosus* (2)										1		1							**64**
	*Lb. curvatus* (1)												1							**64**
	*L. cremoris* (3)										2	1								**32**
	*Lc. cremoris* (3)										1	2								**64**
	*P. pentosaceus* (16)											1	5	10						**64**
	*W. cibaria* (15)									2		7	5	1						**n.a.**
Erythromycin	*Lb. carnosus* (2)				2															**1**
	*Lb. curvatus* (1)				1															**1**
	*L. cremoris* (3)			2	1															**1**
	*Lc. cremoris* (3)			1	2															**1**
	*P. pentosaceus* (16)			1	4	7		3				**1**								**1**
	*W. cibaria* (15)					9	5				1									**n.a.**
Clindamycin	*Lb. carnosus* (2)		1		1															**1**
	*Lb. curvatus* (1)	1																		**1**
	*L. cremoris* (3)	2			1															**1**
	*Lc. cremoris* (3)	2		1																**1**
	*P. pentosaceus* (16)	3	2	7					**1**	**3**										**1**
	*W. cibaria* (15)	2			6	5	1		1											**n.a.**
Tetracycline	*Lb. carnosus* (2)							1	1											**8**
	*Lb. curvatus* (1)							1												**8**
	*L. cremoris* (3)					1	1	1												**4**
	*Lc. cremoris* (3)							1	2											**8**
	*P. pentosaceus* (16)								1		13	**2**								**8**
	*W. cibaria* (15)									15										**n.a.**
Chloramphenicol	*Lb. carnosus* (2)							1	1											**4**
	*Lb. curvatus* (1)								1											**4**
	*L. cremoris* (3)								1	2										**8**
	*Lc. cremoris* (3)								3											**4**
	*P. pentosaceus* (16)							1	5	10										**4**
	*W. cibaria* (15)									15										**n.a.**
Neomycin	*Lb. carnosus* (2)							1		1										**n.a.**
	*Lb. curvatus* (1)									1										**n.a.**
	*L. cremoris* (3)					2		1												**n.a.**
	*Lc. cremoris* (3)					3														**n.a.**
	*P. pentosaceus* (16)					1			9	4	2									**n.a.**
	*W. cibaria* (15)					4		6	4		1									**n.a.**
Penicillin	*Lb. carnosus* (2)								1	1										**n.a.**
	*Lb. curvatus* (1)					1														**n.a.**
	*L. cremoris* (3)					3														**n.a.**
	*Lc. cremoris* (3)				1	2														**n.a.**
	*P. pentosaceus* (16)						7	8	1											**n.a.**
	*W. cibaria* (15)							7	7		1									**n.a.**
Linezolid	*Lb. carnosus* (2)							2												**n.a.**
	*Lb. curvatus* (1)								1											**n.a.**
	*L. cremoris* (3)							1	2											**n.a.**
	*Lc. cremoris* (3)						1	2												**n.a.**
	*P. pentosaceus* (16)								15	1										**n.a.**
	*W. cibaria* (15)								15											**n.a.**
Ciprofloxacin	*Lb. carnosus* (2)								2											**n.a.**
	*Lb. curvatus* (1)										1									**n.a.**
	*L. cremoris* (3)								2	1										**n.a.**
	*Lc. cremoris* (3)								1	2										**n.a.**
	*P. pentosaceus* (16)												16							**n.a.**
	*W. cibaria* (15)									5	10									**n.a.**
Rifampicin	*Lb. carnosus* (2)					1	1													**n.a.**
	*Lb. curvatus* (1)					1														**n.a.**
	*L. cremoris* (3)											1	2							**n.a.**
	*Lc. cremoris* (3)						1	2												**n.a.**
	*P. pentosaceus* (16)							2	13	1										**n.a.**
	*W. cibaria* (15)										12	3								**n.a.**
Trimethoprim	*Lb. carnosus* (2)												1		1					**n.a.**
	*Lb. curvatus* (1)										1									**n.a.**
	*L. cremoris* (3)														3					**n.a.**
	*Lc. cremoris* (3)								1	2										**n.a.**
	*P. pentosaceus* (16)										8	8								**n.a.**
	*W. cibaria* (15)														15					**n.a.**

^a^MICs determined by a VetMIC test. The antibiotic dilution ranges were: 0.03-16 mg/L (ampicillin, clindamycin, penicillin and linezolid), 0.25-128 mg/L (vancomycin and ciprofloxacin), 0.5-256 mg/L (gentamicin, streptomycin and neomycin), 2-1024 mg/L (kanamycin), 0.016-8 mg/L (erythromycin), 0.12-64 (tetracycline, chloramphenicol, rifampicin and trimethoprim). MICs which exceeded the upper or lower limit of the tested range are listed in the next dilution series. MICs higher than the EFSA breakpoints are indicated in bold.

^b^LAB with MICs higher than the EFSA breakpoints are considered as resistant strains [15]. n.r., not required; n.a., not available.

### Detection of antibiotic resistance genes

The non-enterococcal strains showing antibiotic resistances in the VetMIC assays (17 strains) were further submitted to PCR in order to identify the presence of the respective antibiotic resistance genes. The tested strains were the following: *Lb. carnosus* B43 (ampicillin resistant), *P. pentosaceus* TPP3 and SMF120 (tetracycline resistant), *P. pentosaceus* LPP32, LPM83 and B5 (clindamycin resistant), *P. pentosaceus* LPV57 and *W. cibaria* P50, P61, P64, P73, SDM381, SDM389, SMA14 and BCS50 (kanamycin resistant), and *P. pentosaceus* LPM78 and *W. cibaria* SMA25 (kanamycin, erythromycin and clindamycin resistant). Acquired antibiotic resistances likely due to added genes were only found in strains within the genera *Pediococcus* (12.5%) and *Weissella* (6.7%). The genes involved in the horizontal transfer of resistance to tetracycline [*tet*(K)*, tet*(L) and *tet*(M)], kanamycin [*aac(6´ )-Ie-aph(2´ ´ )-Ia*] and erythromycin [*erm*(A), *erm*(B) and *erm*(C)] were not detected. However, *P. pentosaceus* LPM78 and *W. cibaria* SMA25 harboured the erythromycin resistance gene *mef*(A/E). The obtained amplicons were sequenced and found to have 99% homology with the macrolide-efflux protein (*mef*E) gene described for *Streptococcus pneumoniae* and other *Streptococcus* spp. Moreover, *P. pentosaceus* LPM78 and LPM83 harboured the *lnu*(A) gene encoding the lincosamide *O*-nucleotidyltransferase that inactivates lincomycin and clindamycin. Sequencing of both amplicons showed 97% and 93% homology with lincosamide nucleotidyltransferase [*lnu*(A)] gene described for *Staphylococcus haemolyticus* and *S. aureus*, respectively. Nevertheless, *lnu*(B) was not detected in any of the tested strains. With regard to *E. faecium* BNM58, which was phenotypically resistant to erythromycin, none of the respective genes [*erm*(A), *erm*(B), *erm*(C) and *mef*(A/E)] were detected.

## Discussion

In this work, the antimicrobial activity against fish pathogens and the *in vitro* safety of 99 LAB previously isolated from fish, seafood and fish products [14] have been assayed by using microbiological, biochemical and genetic assays in order to identify and select the most suitable candidates to be further evaluated as probiotics for a sustainable aquaculture. LAB are widely known for their ability to inhibit bacterial pathogens by the production of antimicrobial compounds such as organic acids, oxygen peroxide and ribosomally-synthesized peptides referred to as bacteriocins, which constitutes a desirable property for probiotics and a sustainable alternative to antibiotics [9,18]. In this respect, most of the LAB of aquatic origin tested in this work displayed a broad antimicrobial spectrum against the main Gram-positive and Gram-negative fish pathogens, being remarkable that a high number of strains (24 out of 49 strains, 49%) were identified as potential bacteriocin producers. Recently, bacteriocin production ability has been proposed as a key property for selection of probiotic LAB to be used in aquaculture as an alternative to antibiotics to fight against fish pathogen infections [19], similarly as proposed for human and farm animal probiotics [20-22]. In aquaculture farming, lactococcosis produced by the zoonotic agent *L. garvieae*, causing hemorrhagic septicaemia and meningoencephalitis, is one of the most serious diseases affecting several marine and fresh water fish species [23]. With regard to this, our work shows that putative bacteriocinogenic LAB active against this relevant fish pathogen are common amongst the microbiota isolated from aquatic animals (10 strains, 20%).

The application of probiotics in aquaculture may modify the microbial ecology of the aquatic hosts and their surrounding environment, and thus the assessment of their safety to the target aquatic species, the environment and humans constitutes an essential issue [24]. To date, several studies describing the screening and evaluation of LAB as probiotic candidates for aquaculture have been reported [25-28]; however, the safety assessment of the strains is generally limited to *in vivo* challenge tests and rearing trials in order to confirm their lack of toxicity to the aquatic hosts [24,25,28-31]. Strikingly, *in vitro* safety assessment studies have not been generally addressed, despite they have lower economic and ethic costs and result very effective to evaluate the safety of a high number of candidate probiotic strains not only for the host species, but also for humans and the environment. According to EFSA [13], most of the LAB species tested in this work (*P. pentosaceus*, *Lb. curvatus*, *L. lactis*, *Lc. mesenteroides*) are included in the QPS list and, therefore, demonstration of their safety only requires confirmation of the absence of determinants of resistance to antibiotics of human and veterinary clinical significance. However, in the case of enterococci, a more thorough, strain-specific evaluation is required to assess the risk associated to their intentional use in the food chain, while no guidelines are given for the safety assessment of the species *W. cibaria*[13].

Our results show that enterococcal virulence factors were more frequently found in *E. faecalis* than in *E. faecium*, which is in concordance with previous reports [32-34]. In this respect, most of the *E. faecalis* (95%) and a large percentage of the *E. faecium* (53%) strains evaluated in this work showed, at least, one virulence factor, being *efaAfs*, *gelE* and *agg* the most frequently detected genes. With regard to *gelE*, which encodes for an extracellular zinc endopeptidase that hydrolyzes gelatin, collagen, hemoglobin, and other bioactive compounds, this gene was detected at high frequency in *E. faecalis*, with all the *gelE*^*+*^ strains showing gelatinase activity. However, five out of nine *E. faecium* strains harbouring *gelE* were unable to degrade gelatin, suggesting the carriage of a non-functional gene, as previously reported [32,33]. Likewise, in the case of *E. faecium* P68 and *E. faecium* GM29 harbouring *cylL*_*L*_*cylL*_*S*_, the lack of hemolytic activity may be explained by the absence of *cylM*, whose product is involved in the post-translational modification of cytolysin. On the other hand, *esp* and *hyl*, which encode a cell wall-associated protein involved in immune evasion and an hyaluronidase enzyme, respectively, were not found in any of the tested LAB. Previous studies have reported that *esp* and *hyl* are more common in ampicillin-resistant/vancomycin-resistant *E. faecium* (VREF) than in ampicillin-susceptible/VREF strains [35]. In this context, the increase in the incidence of VREF at hospital settings has been attributed mainly to the spread of ampicillin-resistant VREF exhibiting *esp* and/or *hyl*[36,37]. Therefore, the fact that the *E. faecium* strains evaluated in this work lack these genes might be related with their non-clinical origin and absence of ampicillin resistance.

The use and frequent overuse of antibiotics, including those used in human medicine, in fish farming has resulted in the emergence and spread of antibiotic-resistant bacteria in the aquaculture environment. This possesses a threat to human and animal health due to the increase of acquired antibiotic resistance in fish pathogens, the transfer of their genetic determinants to bacteria of terrestrial animals and to human pathogens, and the alterations of the bacterial microbiota of the aquatic environment [11,29]. In our study, the percentage of enterococcal strains showing acquired antibiotic resistance was 68%. Interestingly, the results found in *E. faecium* (71%) and *E. faecalis* (62%) were similar, however, higher percentages of resistance to ciprofloxacin and/or norfloxacin, rifampicin, and glycopeptides were observed in *E. faecalis*. Nevertheless, the occurrence of erythromycin and tetracycline resistance was frequently detected amongst *E. faecium* (45%) but only in one *E. faecalis* strain (5%). In spite of the high prevalence of acquired antibiotic resistance found in enterococci of aquatic origin, they showed low incidence or absence of resistance to the clinically relevant antibiotics vancomycin (8.5%) and ampicillin, penicillin and gentamicin, respectively, which is in agreement with previous studies [33,38]. Moreover, the percentages of strains showing antibiotic resistance in the genera *Weissella*, *Pediococcus* and *Lactobacillus* were 60, 44 and 33%, respectively, while none of the leuconostocs and lactococci showed this phenotype. In this regard, our results indicate that the LAB susceptibility patterns of MIC values to clinically relevant antibiotics are species-dependent, similarly as previously described by other authors [39,40]. Moreover, multiple antibiotic resistance was commonly found in strains within the genus *Enterococcus* (37%), mainly in *E. faecalis*, while being very infrequent in the non-enterococcal strains (5%).

According to EFSA [29], the determination of MICs above the established breakpoint levels, for one or more antibiotic, requires further investigation to make the distinction between added genes (genes acquired by the bacteria via gain of exogenous DNA) or to the mutation of indigenous genes. According to our results, acquired antibiotic resistance likely due to added genes is not a common feature amongst the non-enterococcal LAB of aquatic origin (7.5%). In this respect, this genotype was only found in the genera *Pediococcus* (12.5%) and *Weissella* (6.7%). Although *P. pentosaceus* LPV57 and LPM78 showed resistance to kanamycin (MIC of 128 mg/L), the respective resistance gene *aac(6´ )-Ie-aph(2´ ´ )-Ia* was not found in these strains. Similarly, *P. pentosaceus* TPP3 and SMF120 were phenotypically resistant to tetracycline (MIC of 16 mg/L), but did not contain *tet(K), tet(L)* or *tet(M)*. In this respect, Ammor *et al.*[41] reported that pediococci are intrinsically resistant to the latter two antibiotics, as well as to glycopeptides (vancomycin and teicoplanin), streptomycin, ciprofloxacin and trimethoprim-sulphamethoxazole. Other authors proposed a MIC for tetracycline in pediococci ranging between 8 and 16 mg/L [42], or of 32 mg/L for oxytetracycline in *P. pentosaceus*[17]. The tetracycline breakpoints suggested for pediococci by EFSA are lower than the MICs observed in our work and others [17,42]. On the other hand, the only antibiotic resistance detected in *Leuconostoc* strains was for vancomycin, which is an intrinsic property of this genus. It has been previously reported that *Leuconostoc* strains display poor, if any, resistance to antibiotics of clinical interest [38]. With regard to lactococci, the three *L. cremoris* strains evaluated were susceptible to all the antibiotics; however, relatively high MICs for rifampicin (16–32 mg/L) and trimethoprim (≥ 64 mg/L) were detected. In fact, most lactococcal species are resistant to trimethoprim [41]. As expected, all strains of heterofermentative *Lactobacillus* spp. were resistant to vancomycin but susceptible to the rest of the assayed antibiotics, except *Lb*. *carnosus* B43, which showed the highest MIC for ampicillin and penicillin (MICs of 8 and 4 mg/L, respectively). In this context, the presence of modifications in the low affinity penicillin-binding protein (PBP) that confers resistance to penicillin and β-lactams in *E. faecium* and *Streptococcus pneumoniae*, has been reported [43,44]. Moreover, nine PBPs have been described in *Lb. casei* ATCC 393 [45], which leads us to suggest that a similar mechanism may be also responsible for the ampicillin and penicillin resistance found in *Lb*. *carnosus* B43. The resistance to vancomycin detected in *Pediococcus*, *Leuconostoc* and *Lactobacillus* species in this study might be due to the presence of D-Ala-D-Lactate in their peptidoglycan rather than D-Ala-D-Ala dipeptide [46]. In this context, all tested *W. cibaria* strains showed MICs ≥ 128 mg/L for vancomycin, suggesting that vancomycin resistance is an intrinsic property of this species. In relation to *Weissella* spp., studies on antibiotic resistance profiles are very limited [47] and breakpoints have not been defined by EFSA [15]. In our study, most *W. cibaria* strains showed low MIC values; however *W. cibaria* BCS50 showed relatively high MICs for penicillin (8 mg/L) and kanamycin (64 mg/L), and *W. cibaria* SMA25 showed MICs of 128 mg/L for kanamycin, 8 mg/L for gentamicin, erythromycin and neomycin, and 2 mg/L for clindamycin. Therefore, these two strains were discarded of this study, while *W. cibaria* P50, P61, P64, P73, SMA14, SDM381 and SDM389 were not included in the final selection due to their MICs for kanamycin (32–64 mg/L). According to these results, as a rule of thumb, we propose for *W. cibaria* the breakpoints assigned to *Leuconostoc* spp. by EFSA [15], until further studies establish the wild-type MIC ranges within this species. In spite of that, different MICs for rifampicin and trimethoprim for *W. cibaria* and *Lc. cremoris* were found in this study. The reduced susceptibility of *W. cibaria* towards trimethoprim could indicate an intrinsic resistance to this antibiotic [48]. In our work, the only antibiotic resistance genes found were *mef*(A/E), which encodes a drug efflux pump conferring a low to moderate level of resistance to 14 (erythromycin and clarithromycin)- and 15 (azithromycin)-membered macrolides but not to lincosamide or streptogramin B antibiotics [49], and *lnu*(A), encoding the lincosamide *O*-nucleotidyltransferase that inactivates lincomycin and clindamycin [50]. In this respect, *P. pentosaceus* LPM78 and *W. cibaria* SMA25, displaying erythromycin resistance (MIC = 8 and ≥ 8 mg/L, respectively), carried the gene *mef*(A/E), which can be found in a variety of Gram-positive bacteria, including corynebacteria, enterococci, micrococci, and several streptococcal species [51,52]. On the other hand, two pediococci (*P. pentosaceus* LPM78 and LPM83) that showed resistance to clindamycin (MIC = 4 and 2 mg/L, respectively) carried the gene *lnu*(A), which had been only previously found in staphylococci, streptococci, enterococci and lactobacilli of animal origin and in staphylococci isolated from humans [50,53]. Strikingly, the clindamycin resistant strains *P. pentosaceus* LPP32 and B5 and *W. cibaria* SMA25 (MIC = 4 and 2 mg/L, respectively) did not harbour this gene nor *lnu*(B). To our knowledge, this is the first description of *mef*(A/E) in the genera *Pediococcus* and *Weissella*, and *lnu*(A) in the genus *Pediococcus*. The detection of resistance genes for macrolide and lincosamide in non-enterococcal strains suggests a wider distribution of this group of genes than previously anticipated.

The *in vitro* subtractive screening proposed in this work also include the assessment of bile salts deconjugation, mucin degradation, biogenic amine production and other potentially detrimental enzymatic activities such as the β-glucuronidase activity, which should be absent in probiotic candidates [54-56]. Excessive deconjugation of bile salts may be unfavourable in animal production since unconjugated bile acids are less efficient than their conjugated counterparts in the emulsification of dietary lipids. In addition, the formation of micelles, lipid digestion and absorption of fatty acids and monoglycerides could be impaired by deconjugated bile salts [57]. Similarly, excessive degradation of mucin may be harmful as it may facilitate the translocation of bacteria to extraintestinal tissues [55]. In this respect, it is worthy to note that none of the 49 tested LAB deconjugated bile salts nor exhibited mucinolytic activity, the latter indicating their low invasive and toxigenic potential at the mucosal barrier. These results are in accordance with previous findings showing that LAB do not degrade mucin *in vitro*[58,59]. Moreover, β-glucuronidase activity has been associated with the generation of potential carcinogenic metabolites [56]; however, none of the LAB tested in our study displayed this harmful enzymatic activity. In a previous work [60], we demonstrated that none of the 40 non-enterococcal strains evaluated herein produced histamine, tyramine or putrescine. With regard to enterococci, the nine *E. faecium* strains only produced tyramine, being *E. faecium* CV1 a low producer of this biogenic amine. Although the lack of biogenic amine production by probiotic strains is a desirable trait, it should be borne in mind that tyramine production by enterococci is a very frequent trait [60,61]. Finally, several studies have suggested that probiotic microorganisms might exert a beneficial effect in the digestion process of fish due to the production of extracellular enzymes [62-65]. In our work, the LAB strains of aquatic origin within the genera *Pediococcus*, *Enterococcus* and *Lactobacillus* showed a higher number of enzymatic activities than *Lactococcus*, *Leuconostoc* and *Weissella*, being the enzymatic profiles similar amongst strains within the same genus. In this respect, nearly all the strains produced phosphatases, which might be involved in nutrient absorption [64], and peptidases and glucosidases that breakdown peptides and carbohydrates, respectively. However, the tested LAB showed weak lipolytic activity and no proteolytic activity.

## Conclusions

This work shows that antimicrobial/bacteriocin activity against fish pathogens is a widespread probiotic property amongst LAB isolated from aquatic animals regarded as human food. However, particular safety concerns based on antibiotic resistances and virulence factors were dominant within *E. faecalis* (100%) and *E. faecium* (79%), and acquired antibiotic resistance genes were not commonly found (7.5%; erythromycin and clindamycin) amongst the non-enterococcal isolates of aquatic origin. To our knowledge, this is the first large-scale study describing the antimicrobial activity against fish pathogens and the safety assessment beyond the QPS approach of LAB isolated from aquatic animals. The *in vitro* subtractive screening presented herein, which allowed the selection of 33 strains (8 *E. faecium*, 11 *P. pentosaceus*, 1 *Lb. carnosus*, 1 *Lb. curvatus*, 3 *L. cremoris*, 3 *Lc. cremoris* and 6 *W. cibaria*) out of 99 LAB isolates of aquatic origin, constitutes a valuable strategy for the large-scale preliminary selection of putatively safe LAB intended for use as probiotics in aquaculture and to avoid the spreading of bacterial cultures with harmful traits into the aquatic environment. Nevertheless, a comprehensive *in vivo* assessment of their lack of toxicity and undesirable effects must be also carried out using cell lines, live food and, ultimately, aquatic animals before their unequivocal consideration as safe probiotics for a sustainable aquaculture.

## Methods

### Bacterial strains and growth conditions

A total of 99 LAB (59 enterococci and 40 non-enterococci) of aquatic origin with antimicrobial activity against spoilage and food-borne pathogenic bacteria of concern for the fish industry, previously isolated and identified by our group from fish, seafood and fish products [14], were used in this study (Table 1). The LAB strains were isolated on non-supplemented MRS (Oxoid, Ltd., Basingstoke, United Kingdom) or KAA (Oxoid) agar (1,5%, w/v) at 25°C, and taxonomically identified [14] by sequencing of the genes encoding 16S rRNA (*16S rDNA*) [66] and/or superoxide dismutase (*sodA*) [67]. Unless otherwise stated, LAB were grown aerobically in MRS broth at 32°C.

### Direct antimicrobial activity assay

The antimicrobial activity of the 99 LAB against the main Gram-positive and Gram-negative fish pathogens was assayed by a qualitative stab-on-agar test (SOAT) as previously described by Cintas *et al*. [68]. Briefly, pure cultures were stabbed onto MRS or Tryptone Soya Agar (TSA) (Oxoid) plates supplemented with glucose (2%, w/v) and incubated at 32°C for 5 h, and then 40 ml of the corresponding soft agar (0.8%, w/v) medium containing about 1 × 10^5^ CFU/ml of the indicator strain was poured over the plates. After incubation at 28-37°C for 16–24 h depending on the indicator strain, the plates were checked for inhibition zones (absence of visible microbial growth around the stabbed cultures), and only inhibition halos with diameters >3 mm were considered positive. *L. garvieae* JIP29-99 was grown aerobically in Tryptone Soya Broth (TSB) (Oxoid) at 37°C. *S. agalactiae* CF01173 and *S. iniae* LMG14521 were grown aerobically in Brain Heart Infusion (BHI) broth (Oxoid) at 37°C. *A. hydrophila* CECT5734, *Ls. anguillarum* CECT4344, *Ls. anguillarum* CECT7199, and *Ph. damselae* CECT626 strains were grown aerobically in TSB at 28°C. *V. alginolyticus* CECT521 was grown aerobically in TSB supplemented with NaCl (1%, w/v; Panreac Química S.A.U, Barcelona, Spain) at 28°C.

### Extracellular antimicrobial activity assay

The antimicrobial activity of supernatants from LAB cultures grown in MRS broth at 32°C for 16 h was determined by an agar well-diffusion test (ADT) as previously described by Cintas *et al.*[68]. Supernatants were obtained by centrifugation of cultures at 10,000 × *g* at 4°C for 10 min, adjusted to pH 6.2 with 1 M NaOH, filter-sterilized through 0.22 μm-pore-size filters (Millipore Corp., Bedford, Massachussets, USA) and stored at −20°C until use. Fifty-μl aliquots of cell-free culture supernatants were placed into wells (6-mm diameter) cut in cooled MRS or TSB agar (0.8%, wt/vol) plates previously seeded (1 × 10^5^ CFU/ml) with the indicator microorganisms *Pediococcus damnosus* CECT4797, *L. garvieae* JIP29-99 or *A. hydrophila* CECT5734. After 2 h at 4°C, the plates were incubated under the same conditions mentioned above to allow for the growth of the target microorganisms and then analyzed for the presence of inhibition zones around the wells. To determine the proteinaceous nature of the antimicrobial compounds, supernatants showing antimicrobial activity were subjected to proteinase K treatment (10 mg/ml) (AppliChem GmbH, Germany) at 37°C for 2 h. After proteinase K inactivation by heat treatment (100°C, 10 min), samples were assayed for residual antimicrobial activity by an ADT as described above using *P. damnosus* CECT4797 as indicator microorganism. Supernatants with no added enzyme were treated as indicated above and used as controls. For further characterization of the antimicrobial compounds, 7 ml of supernatants from an overnight culture of LAB were subjected to peptide concentration by ammonium sulphate precipitation. Ammonium sulphate was gradually added to the supernatants to achieve 50% saturation. Samples were kept at 4°C with stirring for 3 h, and then centrifuged at 10,000 × *g* at 4°C for 30 min. Pellets and floating solid material were combined and solubilized in 350 μl of 20 mM sodium phosphate (pH 6.0), and antimicrobial activity of the resulting 20-fold concentrated supernatants was determined by an ADT as described above.

### PCR detection of potential virulence factors in enterococci

Detection of genes encoding potential virulence factors in the 59 enterococci was performed by PCR. The following primer pairs were used: TE3/TE4 for detection of *agg* (aggregation substance), TE9/TE10 for *gelE* (gelatinase), TE34/TE36 for *esp* (enterococcal surface protein), TE5/TE6 for *efaAfs* (*Enterococus faecalis* endocarditis antigen) [32], HYLn1/HYLn2 for *hyl* (hyaluronidase) [35], CYLL_L_–R1/CYLL_S_–R2 for *cylL*_*L*_*–cylL*_*S*_ (cytolysin precursor) [69], and RHCT1/RHCT2 for *cylL*_*L*_*–cylL*_*S*_*-cylM* (cytolysin precursor and posttranslational modifier) [70]. Oligonucleotide primers were obtained from Sigma-Genosys Ltd. (Cambridge, United Kingdom). The positive control strains for detection of potential virulence factors were the following: *E. faecalis* P4 for *cylL*_*L*_*–cylL*_*s*_, *cylL*_*L*_*–cylL*_*S*_*–cylM*, *agg*, *gelE* and *efaAfs*, *E. faecalis* P36 for *esp*[32], and *E. faecium* C68 for *hyl*[35]. PCR-amplifications were performed from total bacterial DNA obtained using the Wizard DNA Purification Kit (Promega, Madrid, Spain) in 25 μl reaction mixtures with 1 μl of purified DNA, 0.7 μM of each primer, 0.2 mM of each dNTP, buffer 1×, 1.5 mM MgCl_2_ and 0.75 U of Platinum Taq DNA polymerase (Invitrogen, Madrid, Spain). Samples were subjected to an initial cycle of denaturation (97°C for 2 min), followed by 35 cycles of denaturation (94°C for 45 s), annealing (48 to 64°C for 30 s) and elongation (72°C for 30 to 180 s), ending with a final extension step at 72°C for 7 min in an Eppendorf Mastercycler thermal cycler (Eppendorf, Hamburg, Germany). PCR products were analyzed by electrophoresis on 1-2% (w/v) agarose (Pronadisa, Madrid, Spain) gels stained with Gel red (Biotium, California, USA), and visualized with the Gel Doc 1000 documentation system (Bio-Rad, Madrid, Spain). The molecular size markers used were HyperLadder II (Bioline GmbH, Germany) and 1Kb Plus DNA ladder (Invitrogen).

### Production of gelatinase by enterococci

Gelatinase production was determined using the method previously described by Eaton and Gasson [32]. Briefly, enterococci were grown in MRS broth overnight at 32°C, and streaked onto Todd-Hewitt (Oxoid) agar plates (1.5%, w/v) containing 30 g of gelatine per litre. After incubation overnight incubation at 37°C, the plates were placed at 4°C for 5 h before examination for zones of turbidity (protein hydrolysis) around the colonies. *E. faecalis* P4 was used as positive control.

### Production of hemolysin

To investigate hemolysin production by the 99 LAB, the strains grown in MRS broth were streaked onto layered fresh horse blood agar plates (BioMérieux, Marcy l'Étoile, France) and grown at 37°C for 1–2 days [32]. β-hemolysis was revealed by the formation of clear zones surrounding the colonies on blood agar plates. *E. faecalis* P4 was used as positive control.

### Determination of antibiotic susceptibility

Antibiotic susceptibility of the 59 enterococci was determined by overlaying antibiotic-containing disks (Oxoid) on Diagnostic Sensitivity Test Agar (Oxoid) previously seeded with approximately 1 × 10^5^ CFU/ml of each enterococcal isolate. The antibiotics tested were ampicillin (10 μg), chloramphenicol (30 μg), ciprofloxacin (5 μg), erythromycin (15 μg), gentamicin (120 μg), nitrofurantoin (300 μg), norfloxacin (10 μg), penicillin G (10 IU), rifampicin (5 μg), teicoplanin (30 μg), tetracycline (30 μg), and vancomycin (30 μg). Inhibition zone diameters were measured after overnight incubation of the plates at 37°C. Resistance phenotypes were recorded as recommended by the Clinical and Laboratory Standards Institute [71]. *E. faecalis* CECT795 and *Staphylococcus aureus* CECT435 were used for quality control. The minimum inhibitory concentration for the 49 pre-selected LAB was determined by a broth microdilution test using e-cocci (for enterococci), and Lact-1 and Lact-2 (for non-enterococcal strains) VetMIC microplates (National Veterinary Institute, Uppsala, Sweden). The antibiotics evaluated for enterococci were ampicillin, vancomycin, gentamicin, kanamycin, streptomycin, erythromycin, tetracycline, chloramphenicol, narasin, and linezolid, while for the non-enterococcal strains, the tested antibiotics were ampicillin, vancomycin, gentamicin, kanamycin, streptomycin, erythromycin, clindamycin, tetracycline, chloramphenicol, neomycin, penicillin, linezolid, ciprofloxacin, rifampicin, and trimethoprim. Individual colonies were suspended in a sterile glass tube containing 5 ml saline solution (0.85% NaCl) to a turbidity of 1 in the McFarland scale (approx. 3 × 10^8^ CFU/ml) and further diluted 1000-fold. Iso-sensitest (IST) broth (Oxoid) was used for enterococci, while LSM medium (IST:MRS, 9:1) was used for all the non-enterococcal strains except *Lactobacillus curvatus* subsp. *curvatus* BCS35, that required LSM broth supplemented with 0.03% (w/v) L-cysteine (Merck KGaA) [72]. Fifty or 100 μl of the diluted enterococcal and non-enterococcal suspensions, respectively, was added to each microplate well which was then sealed with a transparent covering tape and incubated at 37°C for 18 h (in the case of *Lb. curvatus* BCS35, the plates were incubated anaerobically at 32°C for 18 h). After incubation, MICs were established as the lowest antibiotic concentration that inhibited bacterial growth, and interpreted according to the breakpoints identified by the FEEDAP Panel and adopted by EFSA to distinguish between susceptible and resistant strains [15]. Accordingly, strains showing MICs higher than the respective breakpoint were considered as resistant. *E. faecalis* CECT795 and *S. aureus* CECT794 were used for quality control of e-cocci, and Lact-1 and Lact-2 VetMIC microplates, respectively.

### Deconjugation of bile salts

The ability of the 49 pre-selected LAB to deconjugate primary and secondary bile salts was determined according to Noriega *et al.*[73]. Bile salt plates were prepared by adding 0.5% (w/v) sodium salts of taurocholate (TC) and taurodeoxycholate (TDC) (Sigma-Aldrich Corporation, St. Louis, Missouri, USA) to MRS agar (1.5%, w/v) supplemented with 0.05% (w/v) L-cysteine (Merck KGaA, Darmstadt, Germany). Overnight liquid cultures of strains (10 μl) were spotted onto agar plates and incubated under anaerobic conditions (Anaerogen, Oxoid) at 37°C for 72 h. The presence of precipitated bile acid around the colonies (opaque halo) was considered as a positive result. A fresh fecal slurry of a healthy adult horse was used as positive control for bile salts deconjugating activities.

### Degradation of mucin

The capacity of the 49 pre-selected LAB to degrade gastric mucin was determined as described by Zhou *et al.*[58]. Mucin from porcine stomach type III (Sigma-Aldrich Corp.) and agar were added to medium B without glucose at concentrations of 0.5% (w/v) and 1.5% (w/v), respectively. A volume of 10 μl of 24 h viable bacterial cultures was inoculated onto the surface of medium B. The plates were incubated anaerobically at 37°C for 72 h, subsequently stained with 0.1% (w/v) amido black (Merck KGaA) in 3.5 M acetic acid for 30 min, and then washed with 1.2 M acetic acid (Merck KGaA). A discoloured zone around the colony was considered as a positive result. A fresh fecal slurry of a healthy adult horse was used as positive control for mucin degradation ability.

### Determination of enzymatic activities

The APIZYM test (BioMérieux, Montallieu Vercieu, France) was used for determination of enzymatic activities of the 49 pre-selected LAB. Cells from cultures grown at 32°C overnight were harvested by centrifugation at 12,000 g for 2 min, resuspended in 2 ml of API Suspension Medium (BioMérieux) and adjusted to a turbidity of 5–6 in the McFarland scale (approx. 1.5-1.9 × 10^9^ CFU/ml). Aliquots of 65 μl of the suspensions were added to each of the 20 reaction cupules in the APIZYM strip. The strips were incubated at 37°C for 4.5 h and the reactions were developed by addition of one drop each of the APIZYM reagents A and B. Enzymatic activities were graded from 0 to 5, and converted to nanomoles as indicated by the manufacturer´ s instructions.

### PCR detection of antibiotic resistance genes

The presence of genetic determinants conferring resistance to aminoglycosides except streptomycin *aac(6´)-Ie-aph(2´´)-Ia*, to erythromycin *erm*(A), *erm*(B), *erm*(C) and *mef*(A/E)], to tetracycline *tet*(K)*, tet*(L) and *tet*(M)], and to lincosamides *lnu*(A) and *lnu*(B)] was determined by PCR in the LAB strains showing antibiotic resistance by the VetMIC assay. PCR-amplifications and PCR-product visualization and analysis were performed as described above using the following primer-pairs: aacF/aacR for detection of *aac(6´)-Ie-aph(2´´)-Ia*[74], ermAI/ermAII for *erm*(A) [75,76], ermBI/ermBII for *erm*(B) [17], ermCI/ermCII for *erm*(C) [17,77], mef(A/E)I/ mef(A/E)II for *mef*(A/E) [75,76], tetKI/ tetKII for *tet*(K) [17], tetLI/tetLII for *tet*(L) [17,78], tetMI/tetMII for *tet*(M) [17,78], lnuA1/lnuA2 for *lnu*(A) [79], lnuB1/lnuB2 for *lnu*(B) [50]. *E. faecalis* C1570 was used as positive control for amplification of *erm*(C), *lnu*(A) and *tet*(K) and *E. faecalis* C1231 for *erm*(A). *E. faecium* 3Er1 (clonal complex of hospital-associated strain CC9) and *E. faecium* RC714 were used as positive controls for amplification of *aac(6´)-Ie-aph(2´´)-Ia*, *tet*(M*)* and *tet*(L), and for *erm*(B) and *mef*(A/E), respectively. The amplicons obtained with *mef*(A/E) and *lnu*(A) specific primers were purified by using the NucleoSpin Extract II Kit (Macherey-Nagel GmbH & Co. KG, Düren, Germany) and both DNA strands were sequenced at the Unidad de Genómica (Parque Científico de Madrid, Facultad de Ciencias Biológicas, Universidad Complutense de Madrid, Spain). Analysis of DNA sequences was performed with the BLAST program available at the National Center for Biotechnology Information (NCBI).

## Abbreviations

LAB: Lactic Acid Bacteria; FAO: Food and Agriculture Organization of the United Nations; WHO: World Health Organization; EFSA: European Food Safety Agency; QPS: Qualified Presumption of Safety; EC: European Commission; MRS: de Man, Rogosa and Sharpe; KAA: Kanamycin, Aesculin Azide.

## Competing interests

The authors declare that they have no competing interests.

## Authors' contributions

EMA carried out the phenotypic and genetic analyses, prepared the manuscript draft and participated in the design of the experiments. BGS carried out the isolation of the LAB strains and collaborated in the genetic studies. CA contributed to the phenotypic analyses and to prepare the manuscript draft. CC participated in the phenotypic analyses. RC collaborated in the antibiotic susceptibility tests. LMC conceived the study and, together with CH and PEH, designed the experiments, analyzed the results and revised the manuscript. All authors read and approved the final version of the manuscript.

## References

[B1] FAO FAO Fisheries Department. State of world aquaculture 2006 FAO Fish Tech Pap 2006 500 1 134

[B2] FAO Responsible use of antibiotics in aquaculture FAO Fish Tech Pap 2005 469 1 97

[B3] Cabello FC Heavy use of prophylactic antibiotics in aquaculture: a growing problem for human and animal health and for the environment Environ Microbiol 2006 8 1137 1144 10.1111/j.1462-2920.2006.01054.x 16817922

[B4] Austin B The bacterial microflora of fish, revised ScientificWorldJournal 2006 6 931 945 16906326 10.1100/tsw.2006.181 PMC5917212

[B5] Robertson PAW O’Dowd C Burrells C Williams P Austin B Use of Carnobacterium sp. as a probiotic for Atlantic salmon (Salmo salar L.) and rainbow trout (Oncorhynchus mykiss, Walbaum) Aquaculture 2000 185 235 243 10.1016/S0044-8486(99)00349-X

[B6] Wang Y-B Li J-R Lin J Probiotics in aquaculture: challenges and outlook Aquaculture 2008 281 1 4 10.1016/j.aquaculture.2008.06.002

[B7] Defoirdt T Sorgeloos P Bossier P Alternatives to antibiotics for the control of bacterial disease in aquaculture Curr Opin Microbiol 2011 14 251 258 10.1016/j.mib.2011.03.004 21489864

[B8] Verschuere L Rombaut G Sorgeloos P Verstraete W Probiotic bacteria as biological control agents in aquaculture Microbiol Mol Biol Rev 2000 64 655 671 10.1128/MMBR.64.4.655-671.2000 11104813 PMC99008

[B9] Gatesoupe FJ Updating the importance of lactic acid bacteria in fish farming: natural occurrence and probiotic treatments J Mol Microbiol Biotechnol 2008 14 107 114 10.1159/000106089 17957117

[B10] FAO/WHO Probiotics in food. Health and nutritional properties and guidelines for evaluation FAO Food Nutr Pap 2006 85 1 50

[B11] EC On a generic approach to the safety assessment of microorganisms used in feed/food and feed/food production - A working paper open for comment 2003 http://ec.europa.eu/food/fs/sc/scf/out178_en.pdf

[B12] EFSA Introduction of a Qualified Presumption of Safety (QPS) approach for assessment of selected microorganisms referred to EFSA The EFSA Journal 2007 587 1 16

[B13] EFSA Maintenance of the list of QPS biological agents intentionally added to food and feed (2011 update) The EFSA Journal 2011 9 1 82

[B14] Gómez-Sala B Basanta A Sánchez J Martín M Criado R Gutiérrez J Citti R Herranz C Hernández PE Cintas LM Antimicrobial activity of lactic acid bacteria isolated from aquatic animals and fish products13éme Colloque du Club des Bactéries Lactiques, p 45 Abstracts 2004 Nantes, France: ENITIAA and French National Institute for Agricultural Research (INRA)

[B15] EFSA Guidance on the assessment of bacterial susceptibility to antimicrobials of human and veterinary importance EFSA Journal 2012 10 2740 2749

[B16] Collins MD Samelis J Metaxopoulos J Wallbanks S Taxonomic studies on some leuconostoc-like organisms from fermented sausages: description of a new genus Weissella for the Leuconostoc paramesenteroides group of species J Appl Bacteriol 1993 75 595 603 10.1111/j.1365-2672.1993.tb01600.x 8294308

[B17] Klare I Konstabel C Werner G Huys G Vankerckhoven V Kahlmeter G Hildebrandt B Müller-Bertling S Witte W Goossens H Antimicrobial susceptibilities of Lactobacillus, Pediococcus and Lactococcus human isolates and cultures intended for probiotic or nutritional use J Antimicrob Chemother 2007 59 900 912 10.1093/jac/dkm035 17369278

[B18] Ringø E Gatesoupe FJ Lactic acid bacteria in fish: a review Aquaculture 1998 160 177 203 10.1016/S0044-8486(97)00299-8

[B19] Desriac F Defer D Bourgougnon N Brillet B Le Chevalier P Fleury Y Bacteriocin as weapons in the marine animal-associated bacteria warfare: inventory and potential applications as an aquaculture probiotic Mar Drugs 2010 8 1153 1177 10.3390/md8041153 20479972 PMC2866480

[B20] O'Shea EF Cotter PD Stanton C Ross RP Hill C Production of bioactive substances by intestinal bacteria as a basis for explaining probiotic mechanisms: Bacteriocins and conjugated linoleic acid Int J Food Microbiol 2012 152 189 205 10.1016/j.ijfoodmicro.2011.05.025 21742394

[B21] Gillor O Etzion A Riley MA The dual role of bacteriocins as anti- and probiotics Appl Microbiol Biotechnol 2008 81 591 606 10.1007/s00253-008-1726-5 18853155 PMC2670069

[B22] Corr SC Li Y Riedel CU O'Toole PW Hill C Gahan CG Bacteriocin production as a mechanism for the antiinfective activity of Lactobacillus salivarius UCC118 Proc Natl Acad Sci USA 2007 104 7617 7621 10.1073/pnas.0700440104 17456596 PMC1863472

[B23] Vendrell D Balcazar JL Ruiz-Zarzuela I de Blas I Girones O Muzquiz JL Lactococcus garvieae in fish: a review Comp Immunol Microbiol Infect Dis 2006 29 177 198 10.1016/j.cimid.2006.06.003 16935332

[B24] Decamp O Moriarty D Aquaculture species profit from probiotics Feed Mix 2007 15 20 23

[B25] Dimitroglou A Merrifield DL Carnevali O Picchietti S Avella M Daniels C Güroy D Davies SJ Microbial manipulations to improve fish health and production - A Mediterranean perspective Fish Shellfish Immunol 2011 30 1 16 10.1016/j.fsi.2010.08.009 20801223

[B26] Nikoskelainen S Salminen S Bylund G Ouwehand AC Characterization of the properties of human- and dairy-derived probiotics for prevention of infectious diseases in fish Appl Environ Microbiol 2001 67 2430 2435 10.1128/AEM.67.6.2430-2435.2001 11375147 PMC92891

[B27] Balcázar JL Vendrell D de Blas I Ruiz-Zarzuela I Muzquiz JL Girones O Characterization of probiotic properties of lactic acid bacteria isolated from intestinal microbiota of fish Aquaculture 2008 278 188 191 10.1016/j.aquaculture.2008.03.014

[B28] Merrifield DL Dimitroglou A Foey A Davies SJ Baker RTM Bøgwald J Castex M Ringø E The current status and future focus of probiotic and prebiotic applications for salmonids Aquaculture 2010 302 1 18 10.1016/j.aquaculture.2010.02.007

[B29] Das S Ward LR Burke C Screening of marine Streptomyces spp. for potential use as probiotics in aquaculture Aquaculture 2010 305 32 41 10.1016/j.aquaculture.2010.04.001

[B30] Wang Y-B Tian Z-Q Yao J-T Li W Effect of probiotics, Enteroccus faecium, on tilapia (Oreochromis niloticus) growth performance and immune response Aquaculture 2008 277 203 207 10.1016/j.aquaculture.2008.03.007

[B31] Olmos J Ochoa L Paniagua-Michel J Contreras R Functional feed assessment on Litopenaeus vannamei using 100% fish meal replacement by soybean meal, high levels of complex carbohydrates and Bacillus probiotic strains Mar Drugs 2011 9 1119 1132 10.3390/md9061119 21747750 PMC3131563

[B32] Eaton TJ Gasson MJ Molecular screening of Enterococcus virulence determinants and potential for genetic exchange between food and medical isolates Appl Environ Microbiol 2001 67 1628 1635 10.1128/AEM.67.4.1628-1635.2001 11282615 PMC92779

[B33] Gomes BC Esteves CT Palazzo IC Darini AL Felis GE Sechi LA Franco BD De Martinis EC Prevalence and characterization of Enterococcus spp. isolated from Brazilian foods Food Microbiol 2008 25 668 675 10.1016/j.fm.2008.03.008 18541165

[B34] López M Sáenz Y Rojo-Bezares B Martínez S del Campo R Ruiz-Larrea F Zarazaga M Torres C Detection of vanA and vanB2-containing enterococci from food samples in Spain, including Enterococcus faecium strains of CC17 and the new singleton ST425 Int J Food Microbiol 2009 133 172 178 10.1016/j.ijfoodmicro.2009.05.020 19493581

[B35] Vankerckhoven V Van Autgaerden T Vael C Lammens C Chapelle S Rossi R Jabes D Goossens H Development of a multiplex PCR for the detection of asa1, gelE, cylA, esp, and hyl genes in enterococci and survey for virulence determinants among European hospital isolates of Enterococcus faecium J Clin Microbiol 2004 42 4473 4479 10.1128/JCM.42.10.4473-4479.2004 15472296 PMC522368

[B36] Klare I Konstabel C Mueller-Bertling S Werner G Strommenger B Kettlitz C Borgmann S Schulte B Jonas D Serr A Spread of ampicillin/vancomycin-resistant Enterococcus faecium of the epidemic-virulent clonal complex-17 carrying the genes esp and hyl in German hospitals Eur J Clin Microbiol Infect Dis 2005 24 815 825 10.1007/s10096-005-0056-0 16374593

[B37] Werner G Coque TM Hammerum AM Hope R Hryniewicz W Johnson A Klare I Kristinsson KG Leclercq R Lester CH Emergence and spread of vancomycin resistance among enterococci in Europe Euro Surveill 2008 13 1 11 19021959

[B38] Ogier JC Serror P Safety assessment of dairy microorganisms: the Enterococcus genus Int J Food Microbiol 2008 126 291 301 10.1016/j.ijfoodmicro.2007.08.017 17889954

[B39] Danielsen M Wind A Susceptibility of Lactobacillus spp. to antimicrobial agents Int J Food Microbiol 2003 82 1 11 10.1016/S0168-1605(02)00254-4 12505455

[B40] Vay C Cittadini R Barberis C Hernán Rodríguez C Perez Martínez H Genero F Famiglietti A Antimicrobial susceptibility of non-enterococcal intrinsic glycopeptide-resistant Gram-positive organisms Diagn Microbiol Infect Dis 2007 57 183 188 10.1016/j.diagmicrobio.2006.08.014 17049799

[B41] Ammor MS Flórez AB Mayo B Antibiotic resistance in non-enterococcal lactic acid bacteria and bifidobacteria Food Microbiol 2007 24 559 570 10.1016/j.fm.2006.11.001 17418306

[B42] Danielsen M Simpson PJ O'Connor EB Ross RP Stanton C Susceptibility of Pediococcus spp. to antimicrobial agents J Appl Microbiol 2007 102 384 389 17241343 10.1111/j.1365-2672.2006.03097.x

[B43] Klare I Konstabel C Badstübner D Werner G Witte W Occurrence and spread of antibiotic resistances in Enterococcus faecium Int J Food Microbiol 2003 88 269 290 10.1016/S0168-1605(03)00190-9 14597000

[B44] Albarracín Orio AG Piñas GE Cortes PR Cian MB Echenique J Compensatory evolution of pbp mutations restores the fitness cost imposed by beta-lactam resistance in Streptococcus pneumoniae PLoS Pathog 2011 7 e1002000 10.1371/journal.ppat.1002000 21379570 PMC3040684

[B45] Piuri M Sanchez-Rivas C Ruzal SM Cell wall modifications during osmotic stress in Lactobacillus casei J Appl Microbiol 2005 98 84 95 10.1111/j.1365-2672.2004.02428.x 15610420

[B46] Klein G Hallmann C Casas IA Abad J Louwers J Reuter G Exclusion of vanA, vanB and vanC type glycopeptide resistance in strains of Lactobacillus reuteri and Lactobacillus rhamnosus used as probiotics by polymerase chain reaction and hybridization methods J Appl Microbiol 2000 89 815 824 10.1046/j.1365-2672.2000.01187.x 11119156

[B47] Ayeni FA Sánchez B Adeniyi BA de Los Reyes-Gavilán CG Margolles A Ruas-Madiedo P Evaluation of the functional potential of Weissella and Lactobacillus isolates obtained from Nigerian traditional fermented foods and cow's intestine Int J Food Microbiol 2011 147 97 104 10.1016/j.ijfoodmicro.2011.03.014 21482440

[B48] Ayeni FA Adeniyi BA Ogunbanwo ST Tabasco R Paarup T Peláez C Requena T Inhibition of uropathogens by lactic acid bacteria isolated from dairy foods and cow's intestine in western Nigeria Arch Microbiol 2009 191 639 648 10.1007/s00203-009-0492-9 19529917

[B49] Del Grosso M Iannelli F Messina C Santagati M Petrosillo N Stefani S Pozzi G Pantosti A Macrolide efflux genes mef(A) and mef(E) are carried by different genetic elements in Streptococcus pneumoniae J Clin Microbiol 2002 40 774 778 10.1128/JCM.40.3.774-778.2002 11880392 PMC120261

[B50] Bozdogan B Berrezouga L Kuo MS Yurek DA Farley KA Stockman BJ Leclercq R A new resistance gene, linB, conferring resistance to lincosamides by nucleotidylation in Enterococcus faecium HM1025 Antimicrob Agents Chemother 1999 43 925 929 10103201 10.1128/aac.43.4.925 PMC89227

[B51] Roberts MC Sutcliffe J Courvalin P Jensen LB Rood J Seppala H Nomenclature for macrolide and macrolide-lincosamide-streptogramin B resistance determinants Antimicrob Agents Chemother 1999 43 2823 2830 10582867 10.1128/aac.43.12.2823 PMC89572

[B52] Leclercq R Mechanisms of resistance to macrolides and lincosamides: nature of the resistance elements and their clinical implications Clin Infect Dis 2002 34 482 492 10.1086/324626 11797175

[B53] Achard A Villers C Pichereau V Leclercq R New lnu(C) gene conferring resistance to lincomycin by nucleotidylation in Streptococcus agalactiae UCN36 Antimicrob Agents Chemother 2005 49 2716 2719 10.1128/AAC.49.7.2716-2719.2005 15980341 PMC1168647

[B54] Marteau P Gerhardt MF Myara A Bouvier E Trivin F Rambaud JC Metabolism of bile salts by alimentary bacteria during transit in the human small intestine Microb Ecol Health D 1995 8 151 157 10.3109/08910609509140093

[B55] Ruseler-van Embden JG van Lieshout LM Gosselink MJ Marteau P Inability of Lactobacillus casei strain GG, L. acidophilus, and Bifidobacterium bifidum to degrade intestinal mucus glycoproteins Scand J Gastroenterol 1995 30 675 680 10.3109/00365529509096312 7481531

[B56] Heavey PM Rowland IR Microbial-gut interactions in health and disease, Gastrointestinal cancer Best Pract Res Clin Gastroenterol 2004 18 323 336 10.1016/j.bpg.2003.10.003 15123073

[B57] Begley M Gahan CG Hill C The interaction between bacteria and bile FEMS Microbiol Rev 2005 29 625 651 10.1016/j.femsre.2004.09.003 16102595

[B58] Zhou JS Gopal PK Gill HS Potential probiotic lactic acid bacteria Lactobacillus rhamnosus (HN001), Lactobacillus acidophilus (HN017) and Bifidobacterium lactis (HN019) do not degrade gastric mucin in vitro Int J Food Microbiol 2001 63 81 90 10.1016/S0168-1605(00)00398-6 11205957

[B59] Delgado S O'Sullivan E Fitzgerald G Mayo B Subtractive screening for probiotic properties of Lactobacillus species from the human gastrointestinal tract in the search for new probiotics J Food Sci 2007 72 M310 M315 10.1111/j.1750-3841.2007.00479.x 17995611

[B60] Muñoz-Atienza E Landeta G De Las Rivas B Gómez-Sala B Muñoz R Hernández PE Cintas LM Herranz C Phenotypic and genetic evaluations of biogenic amine production by lactic acid bacteria isolated from fish and fish products Int J Food Microbiol 2011 146 212 216 10.1016/j.ijfoodmicro.2011.02.024 21411165

[B61] Ladero V Fernández M Calles-Enríquez M Sánchez-Llana E Canedo E Martín MC Alvarez MA Is the production of the biogenic amines tyramine and putrescine a species-level trait in enterococci? Food Microbiol 2012 30 132 138 10.1016/j.fm.2011.12.016 22265293

[B62] Ringø E Strom E Tabachek JA Intestinal microflora of salmonids: a review Aquac Res 1995 26 773 789 10.1111/j.1365-2109.1995.tb00870.x

[B63] Bairagi A Sarkar Ghosh K Sen SK Ray AK Enzyme producing bacterial flora isolated from fish digestive tracts Aquacult Int 2002 10 109 121 10.1023/A:1021355406412

[B64] Ramirez RF Dixon BA Enzyme production by obligate intestinal anaerobic bacteria isolated from oscars (Astronotus ocellatus), angelfish (Pterophyllum scalare) and southern flounder (Paralichthys lethostigma) Aquaculture 2003 227 417 426 10.1016/S0044-8486(03)00520-9

[B65] Fjellheim AJ Klinkenberg G Skjermo J Aasen IM Vadstein O Selection of candidate probionts by two different screening strategies from Atlantic cod (Gadus morhua L.) larvae Vet Microbiol 2010 144 153 159 10.1016/j.vetmic.2009.12.032 20097491

[B66] Kullen MJ Sanozky-Dawes RB Crowell DC Klaenhammer TR Use of the DNA sequence of variable regions of the 16S rRNA gene for rapid and accurate identification of bacteria in the Lactobacillus acidophilus complex J Appl Microbiol 2000 89 511 516 10.1046/j.1365-2672.2000.01146.x 11021584

[B67] Poyart C Quesnes G Trieu-Cuot P Sequencing the gene encoding manganese-dependent superoxide dismutase for rapid species identification of enterococci J Clin Microbiol 2000 38 415 418 10618129 10.1128/jcm.38.1.415-418.2000 PMC88737

[B68] Cintas LM Rodríguez JM Fernández MF Sletten K Nes IF Hernández PE Holo H Isolation and characterization of pediocin L50, a new bacteriocin from Pediococcus acidilactici with a broad inhibitory spectrum Appl Environ Microbiol 1995 61 2643 2648 7618877 10.1128/aem.61.7.2643-2648.1995 PMC167537

[B69] Gilmore MS Segarra RA Booth MC Bogie CP Hall LR Clewell DB Genetic structure of the Enterococcus faecalis plasmid pAD1-encoded cytolytic toxin system and its relationship to lantibiotic determinants J Bacteriol 1994 176 7335 7344 7961506 10.1128/jb.176.23.7335-7344.1994 PMC197123

[B70] Hickey RM Twomey DP Ross RP Hill C Production of enterolysin A by a raw milk enterococcal isolate exhibiting multiple virulence factors Microbiology 2003 149 655 664 10.1099/mic.0.25949-0 12634334

[B71] CLSI Performance Standards for Antimicrobial Susceptibility Testing: Twenty–first Informational Supplement M100–S21 2011 Wayne, PA, USA: CLSI

[B72] Klare I Konstabel C Müller-Bertling S Reissbrodt R Huys G Vancanneyt M Swings J Goossens H Witte W Evaluation of new broth media for microdilution antibiotic susceptibility testing of Lactobacilli, Pediococci, Lactococci, and Bifidobacteria Appl Environ Microbiol 2005 71 8982 8986 10.1128/AEM.71.12.8982-8986.2005 16332905 PMC1317481

[B73] Noriega L Cuevas I Margolles A Reyes-Gavilán CG Dl Deconjugation and bile salts hydrolase activity by Bifidobacterium strains with acquired resistance to bile Int Dairy J 2006 16 850 855 10.1016/j.idairyj.2005.09.008

[B74] Donabedian SM Thal LA Hershberger E Perri MB Chow JW Bartlett P Jones R Joyce K Rossiter S Gay K Molecular characterization of gentamicin-resistant Enterococci in the United States: evidence of spread from animals to humans through food J Clin Microbiol 2003 41 1109 1113 10.1128/JCM.41.3.1109-1113.2003 12624037 PMC150269

[B75] Sutcliffe J Grebe T Tait-Kamradt A Wondrack L Detection of erythromycin-resistant determinants by PCR Antimicrob Agents Chemother 1996 40 2562 2566 8913465 10.1128/aac.40.11.2562 PMC163576

[B76] Sutcliffe J Tait-Kamradt A Wondrack L Streptococcus pneumoniae and Streptococcus pyogenes resistant to macrolides but sensitive to clindamycin: a common resistance pattern mediated by an efflux system Antimicrob Agents Chemother 1996 40 1817 1824 8843287 10.1128/aac.40.8.1817 PMC163423

[B77] Strommenger B Kettlitz C Werner G Witte W Multiplex PCR assay for simultaneous detection of nine clinically relevant antibiotic resistance genes in Staphylococcus aureus J Clin Microbiol 2003 41 4089 4094 10.1128/JCM.41.9.4089-4094.2003 12958230 PMC193808

[B78] Werner G AWillems RJ Hildebrandt B Klare I Witte W Influence of transferable genetic determinants on the outcome of typing methods commonly used for Enterococcus faecium J Clin Microbiol 2003 41 1499 1506 10.1128/JCM.41.4.1499-1506.2003 12682136 PMC153884

[B79] Lina G Quaglia A Reverdy ME Leclercq R Vandenesch F Etienne J Distribution of genes encoding resistance to macrolides, lincosamides, and streptogramins among staphylococci Antimicrob Agents Chemother 1999 43 1062 1066 10223914 10.1128/aac.43.5.1062 PMC89111

